# Tumoral Metabolism at the Intersection of Oncogene Signaling, Epigenetics and Immunology: Emerging Therapeutic Strategies in Cancer

**DOI:** 10.3390/cimb48070723

**Published:** 2026-07-15

**Authors:** Bhoomendra A. Bhongade, Areeg Anwer Ali, Mohamed El-Tanani, Shakta Mani Satyam, Sirajunisa Talath, Adil Farooq Wali, Syed Arman Rabbani, Walaa Ibraheem, Karolina Hoffmann, Ashot Avagimyan, Ioannis Ilias, Sorina Ispas, Viviana Maggio, Anna Paczkowska, Manfredi Rizzo

**Affiliations:** 1 RAK College of Pharmacy, RAK Medical and Health Sciences University, Ras Al Khaimah 11172, United Arab Emirates; areeganwer@rakmhsu.ac.ae (A.A.A.); eltanani@rakmhsu.ac.ae (M.E.-T.); sirajunisa@rakmhsu.ac.ae (S.T.); farooq@rakmhsu.ac.ae (A.F.W.); arman@rakmhsu.ac.ae (S.A.R.); walaa.ibraheem@rakmhsu.ac.ae (W.I.);; 2 RAK College of Medical Sciences, RAK Medical and Health Sciences University, Ras Al Khaimah 11172, United Arab Emirates; satyam@rakmhsu.ac.ae; 3 Department and Clinic of Internal Diseases and Metabolic Disorders, Poznan University of Medical Sciences, 61-701 Poznań, Poland; karolinahoffmann@ump.edu.pl; 4 Department of Internal Diseases Propedeutics, Yerevan State Medical University After M. Heratsi, Yerevan 0025, Armenia; ashot.avagimyan@meduni.am; 5 Department of Endocrinology, Diabetes and Metabolism, Elena Venizelou Hospital, GR-11521 Athens, Greece; iliasendo@hippocratio.gr; 6 Department of Anatomy, Faculty of General Medicine, “Ovidius” University, 900470 Constanta, Romania; sorina.ispas@365.univ-ovidius.ro; 7 School of Medicine, PROMISE Department of Health Promotion Sciences Maternal and Infantile Care, Internal Medicine and Medicinal Specialties, University of Palermo, 90133 Palermo, Italy; viviana.maggio01@unipa.it; 8 Department of Pharmacoeconomics and Social Pharmacy, Poznan University of Medical Sciences, 61-701 Poznań, Poland; aniapaczkowska@ump.edu.pl

**Keywords:** tumor metabolism, Warburg effect, metabolic reprogramming, therapeutic targeting, mitochondrial dynamics, tumor microenvironment, metabolic heterogeneity

## Abstract

Metabolic reprogramming is a unifying characteristic of cancer and involves orchestrated changes in glucose, amino acid, lipid, and mitochondrial metabolism that go beyond the well-known Warburg effect. Evidence is accumulating that these metabolic states are actively remodeled by oncogene signaling and tumor suppressor loss, allowing cancer cells to sustain anabolic growth, redox homeostasis, and therapeutic stress. This review provides an overview of new findings on tumor metabolism, mechanisms, and the molecular networks governing this reprogramming. We discuss how the major oncogenic pathways, such as MYC, mTOR, HIF, and AMPK, reprogram metabolism using transcriptional, epigenetic, and post-translational control of metabolic flux. A focus is placed on mitochondrial bioenergetics, dynamics, and metabolite signaling such as cancer cell fitness and stress tolerance-defining factors. We also discussed metabolic crosstalk in the tumor ecosystem, including nutrient competition, metabolite coupling, and immunometabolic reprogramming to coordinate metabolism-mediated effects on tumorigenesis and therapeutic response. The review further considers the mechanistic basis for metabolism-targeted therapies, including pathway dependencies, adaptive responses, and micro-environmental context that constrain clinical benefit. Recent innovations such as spatial metabolomics, single-cell metabolic profiling, and systems-level models have unveiled significant intratumoral heterogeneity of metabolism, and they have provided important information about diverse vulnerabilities to therapeutic intervention. Accordingly, understanding the complex crosstalk between these metabolic networks is crucial to rationally designing combination strategies that selectively leverage cancer-specific metabolic liabilities with minimal toxicities against normal tissues.

## 1. Introduction

The theory that tumor cells harbor unique metabolic traits dates back nearly a century to the revolutionary observations by Otto Warburg, who observed that despite normal oxygen levels present in tumor tissues, glucose is preferentially converted to lactate [1]. This metabolic phenotype, since then known as aerobic glycolysis, was paradoxical because it generates much less adenosine triphosphate (ATP) than that produced by mitochondrial oxidative phosphorylation. For decades, this observation was understood as a metabolic consequence of transformation rather than a cause of tumor formation. This perspective has been gradually changing since the end of the last century with progress in molecular genetics and cancer genomics. That oncogenic mutations in major metabolic enzymes such as succinate dehydrogenase, fumarate hydratase, and isocitrate dehydrogenase have been identified supports the notion that key metabolic changes can play a direct role in tumor development [2]. These findings recast metabolism from a byproduct to a cause of malignancy.

Since this early discovery, cancer metabolism has become a rapidly growing field driven by technological advances that allow for comprehensive metabolic investigation. Using stable isotope tracing approaches, dynamic metabolic fluxes that support anabolic growth and redox homeostasis in proliferating cancer cells have been characterized [3]. Concomitantly, high-resolution mass spectrometry-based metabolomics has shown that intracellular metabolite pools are radically reshaped in the context of oncogenic transformation. Crucially, the combination of metabolomics information with genomics and proteomics analyses has elucidated how oncogenic signaling machinery dynamically remodels metabolic networks to sustain cancer cell growth and survival [4]. Recognition that enhanced metabolism is not just supportive of but integral to the transformed malignant phenotype is the basis for altered metabolism now being considered as one of the emerging hallmarks of cancer [5]. Cancer cells have extraordinary biosynthetic needs to support high rates of cell proliferation, which require constant production of nucleotides, amino acids, lipids, and other macromolecular precursors. Meanwhile, tumors grow in the microenvironment featuring changing fluctuations in oxygen content, deficient resources, and an acidic milieu with metabolic stress [6]. Apart from biosynthesis, there is a challenge for malignant cells to maintain redox balance in the face of increased ROS, as well as adapt towards immune pressure and therapeutic stress [7]. These pressures result in a massive rewiring of metabolism that can alter the expression or activity of metabolic enzymes, reprogram the usage and directionality of metabolic pathways, adjust nutrient transporter abundance, and modify preferred sources of carbon and nitrogen. Importantly, cancer metabolism is highly flexible, and tumor cells readily reprogram their metabolic pathways in response to the environment or drug-induced perturbation [8]. Although this adaptability makes therapeutic targeting challenging, it also exposes context-specific vulnerabilities that could be targeted in a clinical setting. With the understanding that metabolic dependencies are actionable vulnerabilities, interest in devising metabolism-targeted anticancer therapies has intensified. In contrast to several molecular targets that are confined to certain mutational subsets, metabolic liabilities are regulated by more general constraints on sustained proliferation and may therefore be applicable in the broader clinical context. As a result, several metabolic pathway-targeting agents have advanced clinical investigation, with preliminary studies highlighting some promising evidence for antitumor activity within specific contexts [9]. However, demonstrating durable clinical benefit from metabolic insights has been difficult to achieve. The metabolic flexibility, which allows tumors to adopt such a deadly phenotype for survival, is the mechanism by which single-pathway inhibition is resisted [8]. In addition to that, growing non-malignant tissues have many of the same metabolism traits as cancer cells, limiting the therapeutic window. Furthermore, intratumoral metabolic heterogeneity necessarily implies that the suppression of a single pathway might selectively clear only a fraction of the malignant cells while enabling metabolically unique subpopulations to survive [10]. These challenges have driven the refinement of new therapeutic approaches, such as combination therapies promoting synthetic lethal interactions, and the implication of tumor-specific metabolic vulnerabilities defined in a genetic or microenvironmental setting [11]. The recognized science of cancer metabolism has been thoroughly addressed elsewhere [4,5].

Instead, in this review, we compiled developments in the field of tumor metabolism with an emphasis on their translational significance. We began by introducing the main aspects of metabolic reprogramming in cancer, going beyond glucose metabolism and focusing on global alterations involving several metabolic pathways. We then covered the molecular controllers of metabolic adaptation, including tumor suppressor loss and oncogenic signaling pathways and epigenetic regulation. Mitochondrial structure and function were one of the focuses, having become a hotspot in leukemia cell fitness and plasticity. We also discuss how metabolic interaction in the tumor microenvironment modulates cancer development, immune responses, and therapeutic response. Finally, we review the status of metabolism-targeted therapies with a focus on successes and remaining challenges as well as emerging opportunities for advances. Further, we focus on a few concepts that shape our current perception of cancer metabolism. These include the pronounced metabolic diversity between and within tumors [10], the broadening of metabolic reprogramming to encompass amino acid, lipid, and one-carbon metabolism in addition to glycolysis [4], and, more recently, dynamic metabolic interplay between cancer cells and their local environment. We also discuss how new technologies, including spatial metabolomics and computational modeling, are revolutionizing our capacity to dissect and target metabolic liabilities in cancer.

## 2. Signatures of Metabolic Reprogramming in Cancer

### 2.1. The Warburg Effect Revisited: Beyond a Single ‘Warburg’s Metabolism’

Glycolysis is a central synthetic hub that provides intermediates to be consumed by several anabolic pathways. Glucose-6-phosphate can also be shunted into the pentose phosphate pathway to generate ribose sugars needed for nucleotide synthesis and NADPH required for these reductive reactions and antioxidant protection. Other glycolytic intermediates also provide carbon skeletons for the synthesis of amino acids and lipids. Crucially, despite being less energetically efficient, glycolysis can generate ATP more rapidly than mitochondrial respiration—a clear advantage for rapidly proliferating cells, where the emphasis may be on speed rather than yield. Tumor lactic acid production, long considered a metabolic waste product, has been adapted to the concept of lactate as a biologically active species in the tumor microenvironment. The export of lactate induces extracellular acidosis, which may promote tissue invasion, angiogenesis, and weakening of the antitumor immunity [12]. These activities jointly define aerobic glycolysis as a multidimensional assembly instead of a metabolic lack. Aerobic glycolysis is arguably the most well-known metabolic hallmark of cancer, but relative requirements for glycolysis can vary dramatically between malignancies or even within different subpopulations of a single tumor, suggesting that while the original Warburg model generally holds true, it represents only part of the metabolic complexity seen in tumors [1,10]. Although some cancers, particularly pancreatic ductal adenocarcinoma and some hematologic malignancies, still maintain a high level of mitochondrial respiration [13], highly glycolytic tumor cells can retain functional mitochondria for additional cellular processes such as biosynthesis, redox homeostasis, and intracellular signaling [5]. This metabolic flexibility allows cancer cells to respond efficiently to changes in nutrient levels and oxygen concentrations in the tumor microenvironment.

It is critical to distinguish two types of metabolic alteration in tumors. The first type consists of a set of broadly general adaptations that include increased nutrient uptake, expanded biosynthetic capacity, and reinforced redox management. These adaptations appear across most proliferating tumors and reflect the generic demands of unrestrained growth. The second consists of specific dependencies that arise from particular genetic lesions or microenvironments, such as 2-hydroxyglutarate production in IDH-mutant tumors, pseudohypoxic HIF stabilization following VHL loss, and arginine auxotrophy in ASS1-silenced cancers. While the universal adaptations explain the sharing of similar malignant metabolic phenotype, the context-specific dependencies reveal vulnerable targets for treatment. Overlooking this difference has contributed to over-generalized expectations of metabolic cancer therapies.

### 2.2. Amino Acid Metabolism: Dependencies Beyond Glucose

While glucose metabolism has taken center stage in the field, aminos are equally essential for taking cancer to warp speed and keeping it there. Of these, glutamine has been identified as an essential nutrient for several tumors, and some cancers exhibit an even greater dependency on glutamine compared with glucose in certain cases, the latter being known as glutaminolytic tumors [14]. Following uptake, glutamine is hydrolyzed to glutamate by the enzyme glutaminase and then metabolized to α-ketoglutarate through both dehydrogenase and transaminase reactions, refilling the tricarboxylic acid (TCA) cycle with intermediates via a process referred to as glutaminolysis [15]. This pathway balances a variety of cellular demands, such as anaplerotic function, nitrogen supply for nucleotide and amino acid biosynthesis, and redox homeostasis by generating NADPH. In most tumor settings, glutamine-derived carbon constitutes the major anaplerotic input for the TCA cycle in those cases where decreased flux along glucose-derived pyruvate oxidation due to increased lactate output is attenuated [16]. In addition to metabolism, glutamine plays a role in cellular redox homeostasis and epigenetics. It is involved in glutathione production, serving as the primary substrate for antioxidant functions, and the availability of α-ketoglutarate will regulate several dioxygenases responsible for DNA and histone demethylation [17]. Other amino acid dependencies additionally emphasize the metabolic heterogeneity of cancer. Serine and glycine also contribute to the one-carbon metabolism necessary for nucleotide biosynthesis and methylation reactions, which are required for cell proliferation [18]. Serine biosynthesis is commonly upregulated in tumors, and associations with sensitivity to serine deprivation have been observed within cancer settings [19]. Levels of available asparagine limit growth in some leukemias, thus explaining the mechanistic basis for L-asparaginase therapy in acute lymphoblastic leukemia [20]. Consistent with this, arginine deprivation approaches are effective in cancers that do not express argininosuccinate synthetase [21], and methionine limitation via dietary or enzymatic means impairs the growth of several cancer types [22]. Collectively, these dependencies emphasize both the regulatory and biosynthetic roles that amino acids play well beyond their role in protein synthesis.

### 2.3. Lipid Metabolism Reprogramming

Lipids are essential for energy production, as signaling molecules, and also as building blocks of cell membranes for cancer cells. In many cancers, de novo lipid synthesis is upregulated even when nutritional lipids are abundant, recapitulating the metabolic reprogramming observed for glucose metabolism [23]. Fatty acid synthase (FASN), the enzyme that catalyzes the final steps of palmitate synthesis, is often upregulated in malignant tissues and correlates with poor prognosis in various types of cancers [24]. Lipid synthesis is upregulated to contribute to an increase in membrane structural components that are necessary for cell division, the generation of lipid-derived signaling molecules that promote survival, and substrates for protein lipidation processes that affect intracellular trafficking and signal transduction. These biosynthetic routes are closely associated with central carbon metabolism. Mitochondrial citrate is then exported to the cytosol, where it is converted by ATP citrate lyase into acetyl-CoA, a substrate for de novo fatty acid synthesis [25]. Acetyl-CoA carboxylase follows that up with malonyl-CoA, which is incorporated consecutively by fatty acid synthase into growing lipid chains. The reductive potential for lipogenesis is predominantly provided by NADPH from the pentose phosphate pathway, in concert with malic enzyme activity, highlighting the crosstalk between glucose and lipid metabolism. A later desaturation by stearoyl-CoA desaturase generates monounsaturated fatty acids, which might change the properties of the membrane and protect against lipotoxic stress [25]. Apart from anabolic-scaling remodeling, cancer cells often modulate lipid catabolism. Fatty acid oxidation acts as an alternate source of energy under fasting or metabolic challenge conditions [26]. Some cancers, such as prostate and some forms of leukemia, have increased requirements for fatty acid oxidation to promote viability [27]. The equilibrium of lipid biosynthesis and catabolism enables tumor cells to fit environmental and therapeutic demands. This has led to lipid metabolic enzymes such as fatty acid synthase and acetyl-CoA carboxylase being considered attractive targets for cancer therapy, with a number of inhibitors now progressing through preclinical and clinical development [24,28].

### 2.4. One-Carbon Metabolism and Nucleotide Synthesis

Proliferation is a highly energy-consuming process that has high demands to produce nucleotides for DNA replication and RNA synthesis. One-carbon metabolism, including the folate and methionine cycles, is centrally implicated in meeting these needs through the generation of activated one-carbon units for purine and thymidylate biosynthesis [29]. One-carbon serine is a major donor through its conversion to glycine by serine hydroxymethyl transferase, yielding 5,10-methylenetetrahydrofolate. This intermediate serves to fuel the production of purine and thymidylate necessary for genome duplication. De novo serine synthesis is upregulated in many tumors by shunting glycolytic intermediates into the serine biosynthesis pathway through 3-phosphoglycerate, mediated by the enzyme’s phosphoglycerate dehydrogenase, phosphoserine aminotransferase, and phosphoserine phosphatase [17]. Phosphoglycerate dehydrogenase amplification has been reported in subsets of breast cancer and melanoma, with high-level expression associated with unfavorable clinical outcomes [30]. Besides serving as a precursor of nucleotide biosynthesis, serine-fueled one-carbon metabolism also generates NADPH via methylenetetrahydrofolate dehydrogenase and thus connects these pathways to cellular redox status. Furthermore, the spatial separation of one-carbon metabolism into mitochondrial and cytosolic pools adds even more regulatory complexity [31]. Mitochondrial reactions produce formate that may be exported to the cytosol for anabolic purposes. The relative contribution of each compartment differs among the tumor types and is determined by microenvironmental factors such as oxygen availability. In one-carbon metabolism therapeutics, one-carbon metabolism is the target of therapy with a long clinical history, methotrexate being the prototypical antifolate agent. Recent efforts are focused on developing more selective inhibitors and finding predictive biomarkers to increase therapeutic responses [30,32].

### 2.5. Spatial and Temporal Metabolic Heterogeneity

The recognition of spatial and temporal metabolic heterogeneity of tumors has been one of the most profound insights in cancer metabolism [10]. The spatial heterogeneity is due to irregular vascularization and nutrition supply, which results in metabolic niches within the tumor. Cells within close proximity to the blood vessels are relatively well oxygenated and nourished, while cells in poorly perfused areas may be challenged with hypoxia and lack of nutrients. These gradients give rise to different metabolic phenotypes in the two distinct hypoxic environments: the well-oxygenated region supporting oxidative metabolism and the hypoxic zone requiring glycolysis and reductive pathways [6]. This metabolic flexibility, allowing survival under a wide range of environments, also leads to resistance against metabolism-directed therapies [8]. Temporal diversity adds to this complexity as cancer cells dynamically rewire their metabolism to respond to adaptive changes in the environment, therapeutics, and progression of disease [33]. Primary tumors and metastatic lesions have distinct metabolic requirements. Furthermore, metastasizing tumor cells differ fundamentally from dormant tumor cells, highly metastatic tumor (HMT) populations and actively proliferating metastatic cells. Tackling this new metabolic backdrop is an important health concern. However, these complexities are starting to be resolved by recently developed methodological improvements. Single-cell and spatial metabolomics methodologies have demonstrated remarkable intraclonal metabolic heterogeneity even between genetically related cells located within the same microenvironmental compartment [3]. This variation may be due to a combination of stochastic processes and finely tuned responses to minor environmental variations. Metabolic plasticity and metabolic heterogeneity in the tumor microenvironment are emerging as a frontier in cancer research for both tumor evolution and how tumors develop resistance to treatments, as well as their capacity to metastasize. From a therapeutic point of view, our results predict that achieving sustained metabolic targeting likely requires drug combinations that concurrently induce multiple defects in the metabolism [11]. Table 1 describes the metabolic heterogeneity across major cancer types.

Cancer classifications denote the dominant or most frequently reported phenotypic features, serving as diagnostic guidelines rather than as fixed tumor-level properties since substantial intratumoral, interpatient, and intersubtype metabolic heterogeneity exists in most cases. The most notable variation is with glioblastoma where it demonstrates marked intratumoral metabolic heterogeneity across perinecrotic, infiltrative, and IDH-mutant regions. Similarly, acute myeloid leukemia consists of metabolically distinct subpopulations, with leukemic stem cells depending more heavily on OXPHOS than the broader tumor cell population. Renal cell carcinoma further underscores this diversity, with metabolic profiles fluctuating distinctly based on histological subtype and molecular background (clear cell versus papillary and chromophobe tumors).

## 3. Molecular Regulators of Tumoral Metabolism

### 3.1. Oncogene-Driven Metabolic Reprogramming

**Figure 1:**
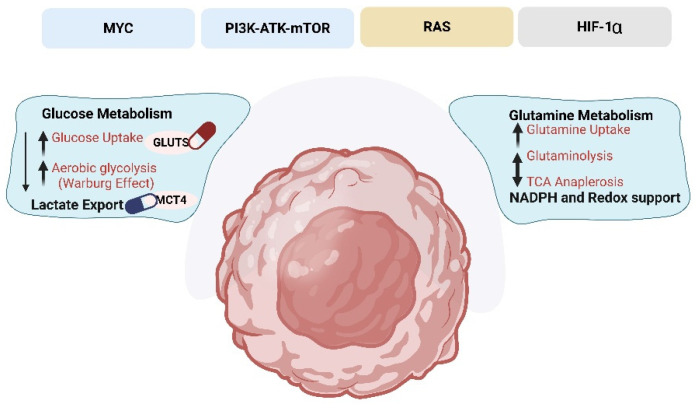
MYC-driven metabolic network and principal therapeutic intervention points. MYC coordinates upregulation of glucose uptake (GLUT transporters), aerobic glycolysis (HK2, PKM2, LDHA), lactate export (MCT4), glutamine uptake and catabolism (GLS, GDH), TCA anaplerosis, nucleotide biosynthesis (SHMT, DHFR), and lipid synthesis (FASN, ACC), creating an integrated anabolic state supporting malignant proliferation. Key pharmacological targets at each node are indicated. *Created in BioRender. Wali, A. F. (2026). BioRender.com/o4kmmuy.*

Emerging consensus in cancer biology suggests that oncogenic signaling and metabolic reprogramming are interwoven events. Metabolic adaptation and genetic transformation do not develop simultaneously but instead occur in concert as oncogenic drivers actively enforce the metabolic changes mediating malignant expansion [43]. This close association explains how otherwise disparate metabolic phenotypes are so consistently evident across different genetic backgrounds of tumors. The myelocytomatosis (MYC) oncogene is a prototype of a one-oncogene mediating energy metabolism reprogramming at multiple layers. Being a universal transcription factor, MYC directly represses many classes of metabolic enzyme and transporter-encoding genes in various pathways. Activation of MYC results in upregulation of glucose transporters and several rate-limiting glycolytic enzymes and thus facilitates heightened glucose uptake accompanied by rapid glycolysis to support the bioenergetic requirements for rigorous proliferation. Meanwhile, MYC induces glutamine metabolism by inducing the expression of several genes, including those encoding glutamine transporters and glutaminase, which support the strong reliance on (i.e., “addiction” to) glutamine metabolism observed in many Myc-dependent cancers [40]. In addition to its regulation of those genes involved in energy production through direct transcriptional mechanisms, MYC has more general effects on the cellular metabolic machinery by initiating mitochondrial biogenesis and ribosomal synthesis and by promoting cell-growth programs. Together, these effects demonstrate how MYC orchestrates a multilayered metabolic program joining glucose, glutamine and nucleotide metabolism in the service of oncogenic transformation. MYC-driven metabolic pathways are depicted in Figure 1.

A further key link between oncogenic signaling and metabolic reprogramming is PI3K/AKT/mTOR [43]. AKT activation increases glucose influx by increasing translocation of glucose transporters to the plasma membrane and activates glycolysis by phosphorylation-dependent kinase activation, including hexokinase and phosphofructokinase. mTOR acts as a key downstream sensor for both nutrient and growth factor signaling, measuring the availability of proteins, lipids and nucleotides according to cellular growth demands [41]. mTOR signaling is the axis of anabolic and catabolic pathways, autophagy being one of the latter [11]. Due to the frequent activation of PI3K/AKT/mTOR signaling in cancer through PIK3CA mutation, PTEN loss or receptor tyrosine kinase signaling, this axis is a central driver for the anabolic metabolic state of proliferating tumor cells. Oncogenic rat sarcoma (RAS) proteins, the most frequently mutated drivers in human cancers, also heavily influence metabolic networks [33]. Mutant RAS stimulates uptake of glucose and flux through glycolysis, as well as glutamine metabolism and macropinocytosis, a parallel nutrient scavenging process. Tumors driven by RAS, for example, are often more dependent on autophagy to recycle material within the cell and maintain biosynthesis when under metabolic stress [11]. The metabolic consequences of these events are transmitted through several downstream signaling cascades, such as RAF/MEK/ERK and PI3K/AKT; this complexity highlights the multiple pathways by which oncogenic signals can be translated into altered metabolism.

### 3.2. Tumor Suppressor Loss and Metabolic Deregulation

Similarly to oncoproteins that activate oncogenes and actively rewire metabolic networks, pernicious loss of tumor suppressor function can drive instigating changes in cell-intrinsic metabolic states. The tumor suppressor p53, being mutated in over half of all human cancers, has pervasive regulation of metabolism not restricted to its classic function in cell cycle arrest and apoptosis. Under basal conditions, p53 inhibits glycolysis and promotes mitochondrial oxidative metabolism by modulating the transcription of metabolic genes [44]. A key mechanism is through upregulation of TP53-induced glycolysis and apoptosis regulator (TIGAR), which lowers cellular fructose-2,6-bisphosphate levels and consequently blunts glycolytic flux, shunting the glucose-derived carbon backbone towards the pentose phosphate pathway to fuel NADPH generation. Loss of p53 eliminates this metabolic restriction, allowing for increased glycolysis and decreased oxidative phosphorylation, leading to a strengthening of Warburg-like attributes. p53 also exerts control on glutamine metabolism by the regulation of glutaminase 2 and regulates lipid metabolism through targeting fatty acid oxidation and lipid synthesis pathways. Furthermore, p53 also regulates redox status and metabolic stress adaptation of the cells [45]. Although p53 loss has been shown to induce metabolic vulnerabilities, such as dependence on glycolysis, it also instills metabolic flexibility that can support the resistance of tumors towards therapeutics. Other tumor suppressors also mold cancer metabolism. Deletion of PTEN leads to persistent activation of PI3K/AKT signaling and subsequent metabolic alterations that support increased levels of anabolism. LKB1, a tumor suppressor with frequent inactivation in lung cancer and other cancers, controls cellular energy sensing primarily via the activation of AMP-activated protein kinase (AMPK). LKB1 loss of function neuters the cellular response to energetic stress, allowing proliferation under nutrient-deprived conditions. RB (retinoblastoma protein) also plays a role in metabolic regulation, which impacts mitochondrial function and nucleotide synthesis [46]. These examples taken together indicate that tumor suppressor loss, in the same way as oncogene activation, is a direct driver of those metabolic phenotypes that distinguish cancer cells.

### 3.3. Response to Hypoxia and HIF-Dependent Metabolic Adaptation

With the outgrowth of tumors beyond their blood supply, hypoxic areas develop, leading to major metabolic stress. Hypoxia response is largely coordinated by hypoxia-inducible factors (HIFs), transcription factors that are stabilized in low-oxygen conditions to engage large-scale metabolic reprogramming programs [6], the most prominent target of which, HIF-1α, activates expression of glucose transporters and almost all glycolytic enzymes, dramatically enhancing glycolytic flux. Simultaneously, HIF-1α suppresses mitochondrial oxygen consumption by activating pyruvate dehydrogenase kinase and thus suppressing pyruvate dehydrogenase activity, limiting the input of pyruvate into the TCA cycle. This concerted change restrains oxidative metabolism to allow ATP production with hypoxia. Metabolic regulation by HIF is not limited to glycolysis. Signaling via HIF regulates amino acid metabolism, including pathways involved in glutamine use and proline synthesis, as well as affecting lipid metabolism by inducing lipid storage and suppressing fatty acid oxidation. In addition, HIF also controls cell homeostatic autophagy and mitophagy, which is required for energy metabolism in the absence of oxygen [47]. These integrated responses reflect the evolutionary function of HIF as a master switch to cell survival under hypoxia. Crucially, the activation of HIF in cancer is not only governed by oxygen levels. Oncogenic and tumor suppressive signaling can serve to stabilize HIF under normoxic conditions by perturbing their regulatory apparatus. Mutations in tricarboxylic acid cycle enzymes, including succinate dehydrogenase and fumarate hydratase, result in accumulation of succinate or fumarate, which inhibit HIF prolyl hydroxylases and thus stabilize HIF [48]. In a similar manner, inactivation of the von Hippel–Lindau (VHL) tumor suppressor leads to HIF stabilization, except loss contributes [37]. This pseudo-hypoxic profile causes metabolic reprogramming even in oxygenated tumor areas and proves how genetics, but also environmental factors, work together to shape the metabolism of tumors.

### 3.4. Epigenetic Control of Metabolic Gene Expression

Metabolism–epigenetics crosstalk has been recognized as one of the important axes in cancer biology. Metabolic pathways produce vital substrates for epigenetic alteration, such as S-adenosylmethionine and acetyl-CoA, which are involved in methylation reactions and histone acetylation, respectively [49]. On the other hand, since transcription of metabolic enzymes is also epigenetically regulated, feedback loops are formed to connect metabolic state to gene expression programs. It was with the finding that isocitrate dehydrogenase (IDH) mutations cause glioma and acute myeloid leukemia by generating the oncometabolite 2-hydroxyglutarate that much of the interest in metabolism-mediated epigenetic modification was stimulated [17]. 2HG is an inhibitor of α-ketoglutarate-dependent dioxygenases, such as TET DNA demethylases and Jumonji-domain histone demethylases, which can result in changes in the chromatin structure and ultimately gene expression [15]. The same process works in tumors with succinate dehydrogenase or fumarate hydratase mutations, resulting in an accumulation of succinate or fumarate and the inhibition of the same class of epigenetic enzymes [48]. In addition to these mutation-mediated transformations, more subtle alterations in the availability of metabolites can also reconfigure epigenetic landscapes. Changes in acetyl-CoA levels directly influence global histone acetylation, and changes in S-adenosylmethionine impact DNA and histone methylation patterns [50]. Metabolites like α-ketoglutarate, succinate, fumarate and lactate are capable of directly influencing the activity of chromatin-modifying enzymes [51]. Such examples of metabolic–epigenetic crosstalk represent potential therapeutic targets where targeted metabolic manipulation could reprogram epigenetic landscapes, restore differentiation or overcome resistance to treatment [34,52]. The key metabolites that are cofactors or inhibitors for epigenetic enzymes are listed in Table 2.

### 3.5. Metabolic Rheostats: Post-Translational Modifications

The main function of protein PTMs is to modulate the abundance, activity and interaction network of proteins in response to stress and changing nutrient conditions. Post-translational modifications offer immediate, reversible mechanisms for tuning metabolic enzyme activity in response to dynamic changes in cellular status. Post-translational modifications, including phosphorylation, one of the most well-studied modifications in biology, regulate the activity of many metabolic enzymes, such as those involved in rate-limiting steps of glycolysis, the TCA cycle and biosynthetic pathways, etc. Oncogenic activation, as well as growth factor signaling, frequently modulates cellular metabolism by the direct phosphorylation of metabolic enzymes by oncogene-associated kinases, including AKT. By contrast, metabolic stress induces stimulation of AMPK and phosphorylation of metabolic enzymes to return energy homeostasis [53]. Acetylation is currently one of the most critical posttranslational modifications in metabolism. Mitochondrial proteins are known to be heavily modified by lysine acetylation, a posttranslational modification that can have profound effects on enzymatic activity and protein stability. The acetylation alteration is also subject to dynamic control by NAD^+^-dependent deacetylases, sirtuins that remove acetyl groups according to cellular redox and energy level [45]. Hence, the acetylation status of metabolic enzymes represents a direct molecular mechanism by which shifts in NAD^+^ availability may be translated into changes in mitochondrial metabolism. Members of the histone deacetylase (HDAC) class, independent of sirtuins, are other proteins involved in controlling metabolic enzyme activity through a deacetylation reaction. Very importantly, the acetylation of metabolic enzymes is not always enzyme-catalyzed. This non-enzymatic acetylation process can proceed as a result of the intrinsic reactivity of acetyl-CoA, thus establishing a direct biochemical connection between cellular metabolite levels and protein modification [38]. Thus, the metabolic state can be stamped on the proteome without transcriptional regulation. In addition to acetylation, a plethora of post-translational modifications, including methylation, ubiquitination, succinylation, and lactylation, orchestrate metabolic enzyme function, localization, and stability [51]. Rapid, fine-tuned control of metabolic flux is made possible by the reversible and diversified nature of these modifications. In cancer, the deregulation of enzymes that add or remove these modifications can even be a driver of metabolic rewiring per se. For instance, changes in sirtuin expression/activity influence the mitochondrial metabolism and cellular stress response. The author’s work is motivated by the central question of how post-translational regulation interacts with genetic and transcriptional mechanisms in the control of tumor metabolism, which continues to be an area under intensive study.

## 4. Mitochondrial Dynamics and Dysfunction

### 4.1. Mitochondrial Metabolism and Biogenesis in Cancer

Early conceptions of cancer metabolism, predominantly based on glycolysis, have overlooked the central role of mitochondria in transformed cells. It is now evident that the majority of tumor cells maintain functional mitochondria, which are essential for cell fitness and survival [5]. Even in cancers with high glycolytic activity, mitochondrial respiration is retained as a source of ATP production (and biosynthetic intermediates through the TCA cycle) and for redox homeostasis. The relative reliance on glycolysis versus oxidative phosphorylation varies to a large extent between cancer types and even within tumors, owing in part to intrinsic genetic programs as well as adaptation based on microenvironment conditions [13]. The mitochondrial biogenesis is tightly controlled by transcriptional co-activator PGC-1α and its related family members, which coordinate nuclear and mitochondrial gene expression to guarantee correct formation of the respiratory complexes. Changes in PGC-1α levels or activity are detected in various malignancies, and some tumors take advantage of increased mitochondrial biogenesis to meet high energetic as well as anabolic needs [55]. Oncogenic MYC positively regulates mitochondrial biogenesis as a centerpiece of its more general program for promoting growth, and other oncogenic pathways like RAS signaling also have the same effect on mitochondrial mass and function [56]. The balance between glycolytic and oxidative metabolism in tumor cells is subject to regulation by multiple parameters, including oncogenic mutations, oxygen and nutrient availability, as well as the stage of the tumor. Some cancers, including certain types of leukemias and pancreatic tumors, exhibit an increased dependence on OXPHOS, which can in turn be therapeutically targeted [13,39]. Interestingly, in glycolysis-orientated tumors, certain cell populations, including cancer stem-like cells, may rely dominantly on mitochondrial respiration [57]. Such intratumoral metabolic diversity could have important implications for therapy, because treatments can differ in their killing potential towards various subpopulations depending on which metabolic pathway they target. Understanding how pathway choice is determined still represents a major challenge in the field.

### 4.2. Fission, Fusion, and Quality Control

**Figure 2:**
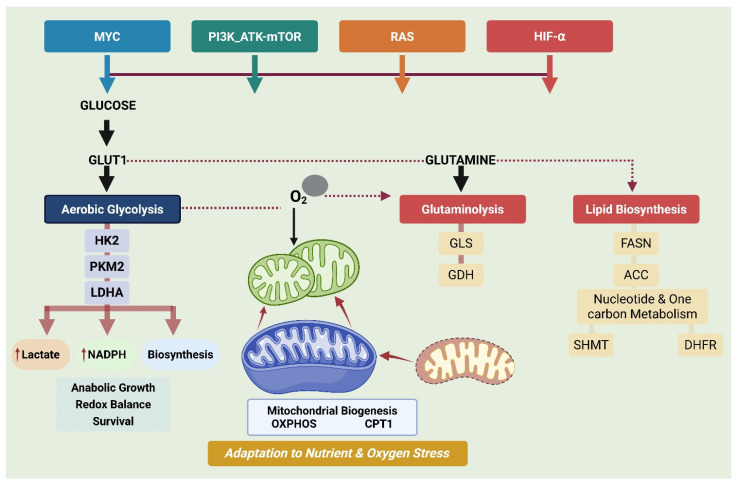
Proposed mechanisms of mitochondrial heterogeneity and intercellular mitochondrial transfer in the tumor microenvironment. The schematic summarizes putative routes by which functional mitochondria may be transferred from stromal cells (MSCs, CAFs, endothelial cells) to metabolically compromised cancer cells via tunnelling nanotubes, extracellular vesicles, and cell fusion, with proposed restoration of OXPHOS capacity and a suggested contribution to chemoresistance. The reverse transfer of damaged mitochondria from cancer to stromal cells is also depicted. The supporting evidence derives predominantly from in vitro and selected in vivo models; the indicated molecular targets of the transfer machinery are therefore based on an exploratory concept and have not been clinically validated. *Created in BioRender. Wali, A. F. (2026). BioRender.com/o4kmmuy.*

Mitochondria are a dynamic network of organelles that constantly move apart from and fuse together with each other, events that promote their function, distribution and integrity. Division is mainly governed by the dynamin-related GTPase DRP1, and fusion is regulated by the mitofusins (MFN) and OPA1 (inner membrane protein) [58]. The interplay of these two processes determines mitochondrial bioenergetics capacity, calcium handling and the tendency to undergo apoptosis. In many cancers, mitochondrial dynamics are deranged, and often, fission becomes dominant with fragmented mitochondria networks [59]. The effects of modulating mitochondrial dynamics in cancer are context-specific. Increased fission may accelerate cell division by allowing efficient mitochondrial inheritance during mitosis. Dysfunctional mitochondria may also be less efficient at using oxidative phosphorylation for producing ATP yet support anabolic metabolism by redistributing and compartmentalizing metabolites. Mitochondrial fission is also crucial for mitophagy, which results in the segregation and elimination of defective mitochondrial entities when cells are stressed [47]. In contrast, fusion mitochondrial function complementation and mitochondrial dysfunction thereby exchange materials among linked mitochondria. As fission and fusion form the basis of mitochondrial health, these proteins controlling their dynamics have been suggested as potential therapeutic targets. DRP1 has also been suggested as a novel and interesting target because the pharmacological inhibition of this protein is able to block tumor progression in preclinical models, although there are still unknowns about how it works [59]. Aberrant mitochondrial dynamics also modulate susceptibility to chemotherapy and targeted agents [10,11], and there is even some evidence that fused mitochondrial networks enhance a cell’s resistance to drug-induced apoptosis. The crosstalk among mitochondrial dynamics, metabolism, and therapeutic response presents an area of great potential for future study. A schematic representation of mitochondrial heterogeneity and intercellular transfer in the TME is shown in Figure 2.

### 4.3. Intercellular Mitochondrial Transfer and Therapeutic Resistance

A new paradigm, the different methods by which mitochondria can be transferred among cells, has been summarized above. In this section, we provide an overview of the key processes for which evidence is emerging. It should be emphasized that although these observations are striking, the evidence base relies mostly on in vitro co-culture systems and limited in vivo models. Consequently, intercellular mitochondrial transfer therefore should be viewed as an emerging and still exploratory concept rather than a validated mechanism of clinical resistance. One of the most surprising developments in modern cancer research is the realization that mitochondria can be transferred between cells with profound implications for tumor bioenergetics and treatment response [60]. Tumor cells are reported to receive mitochondria from the surrounding stroma, including MSCs, endothelial cells and immune cells via routes such as TnTs, EVs, and cell fusion [61,62]. Intercellular transfer of mitochondria may rescue mitochondrial function in metabolically challenged cancer cells and restore oxidative functionality as well as drive resistance to agents targeting mitochondrial metabolism [63]. The implications are broader than that with mitochondrial transfer. Functional mitochondria are transferred from beclin1 (-/-) cells to recipient cells with the transfer of mitochondrial DNA and are capable of impacting metabolic phenotype, as well as the response to stress. Experimental studies have demonstrated that cancer cells take up stromal mitochondria to promote chemoresistance, maintain metastatic capacity and augment stem-like features [63,64]. On the other hand, cancer cells may transfer damaged mitochondria to stromal cells that alter the metabolic environment of the tumor to become pro-tumorigenic. Despite increasing attention, the molecular mechanisms that control mitochondrial transfers (donor–recipient choice and direction of transfer) are still poorly understood. Cellular stress such as hypoxia or chemotherapeutic agents induce mitochondrial transfer, indicating that this process is an adaptive strategy to metabolic challenge [65]. Therapeutically, mitochondrial transfer can be a resistance mechanism that counters the effectiveness of metabolic therapies. In the future, it is possible that therapeutic intervention targeting the machinery responsible for mitochondrial transfer or selectively eliminating cells that accept foreign mitochondria from CD47low BMDMs may provide new treatment opportunities.

### 4.4. Mitochondrial DNA Mutations and Heteroplasmy and cGAS-STING Activation

Mitochondrial DNA (mtDNA) is a small circular genome that encodes 13 subunits of the electron transport chain as well as rRNAs and tRNAs. However, combined with the fact that mtDNA is less protected against reactive oxygen species and has limited capability for DNA repair, this causes a higher rate of mutations in mtDNA than in nuclear DNA [66]. The frequency of occurrence of mtDNA mutations is considerable and can range from point mutations to large deletions, and the variants occur along with high levels of heteroplasmy, where wild-type and mutant forms coexist within a single cell [67]. Their metabolic effects are a function of their functional severity, frequency and the cellular environment in which they occur. Although a severe defect in ETC could lead to an inhibition of oxidative phosphorylation and stimulation of glycolytic dependency, the association between mtDNA mutations and cancer metabolism can be more complex. Some of the mtDNA mutations that cause tumorigenesis impact the bioenergetic system indirectly, via ROS signaling effect, calcium homeostasis or metabolite production, rather than through direct bioenergetic failure. The observations that there is evidence of positive selection for some mtDNA mutations during tumor progression indicate that their context-specific fitness affectivity varies from beneficial to neutral [68]. Therapeutically, mtDNA mutations could define metabolic liabilities. Other tumors showing defective oxidative phosphorylation because of reduced reaction activity (in the case of FH-deficient cells) may show increased sensitivity to inhibition of glycolysis as well, because this would limit metabolic flexibility [43]. In turn, tumors of this type may be resistant to therapies that inhibit mitochondrial respiration. Heteroplasmy is complicated by its dynamic nature: the percentage of mutant mtDNA varies in response to selective pressures, such as anticancer therapies [69]. The role of mtDNA mutations in regulating metabolic dependencies and responses to therapeutic interventions is a critical frontier.

### 4.5. Reactive Oxygen Species: Signaling, Liability and Therapeutic Context

From signaling molecules to bioenergetic modulators, mitochondrial electron transport chain (ETC)-derived reactive oxygen species (ROS) have recently gained extensive attention as important signaling molecules in cancer [10]. While excessive ROS mediate oxidative damage to DNA, proteins and lipids, moderate levels of ROS serve as second messengers governing signaling cascades, transcriptional programs and the activity of metabolic enzymes. Cancer cells generally exhibit increased baseline ROS levels compared to normal cells due to metabolic demands, oncogenic signals and mitochondrial changes. Such heightened oxidative status may have liabilities, as well as adaptive benefits. For resistance against cytotoxic conditions, cancer cells are dependent on antioxidant mechanisms such as glutathione, thioredoxin and superoxide dismutase. This reliance generates therapeutic opportunities, as elevating ROS beyond this is even accompanied by cellular buffering capacity; the latter can reach a limit too and initiate cell death [66]. Several agents are used in chemotherapy and radiotherapy based on capitalizing on this weakness through triggering oxidative stress. Concurrently, ROS induce adaptive signaling cascades to support tumor survival and spread. Oxidative signals stimulate transcription factors, such as NRF2 and HIF, to activate antioxidant- and metabolic-associated genes that contribute to stress resistance [70]. ROS also promote genomic instability, promote tumor evolution, and modulate immune and stromal cell function in the TME [10]. These context-specific effects have confounded therapeutic interventions designed to alter ROS levels. The value of strategies that increase or inhibit ROS should depend on tumor type and genetic background and the environment/agents combined with these other treatments.

## 5. Tumor Microenvironment and Immunometabolism

### 5.1. Metabolic Rivalry and Communal Living in the TME

Tumors are complex systems consisting not only of cancerous cells but also other non-cancerous types of cellular elements such as fibroblasts, endothelial cells, immune cells and the extracellular matrix. These components collectively make up the tumor microenvironment, where metabolic crosstalk stands at the center of tuning tumor behavior [71]. This environment is also heavily remodeled by proliferating cancer cells, which have high metabolic needs and consume substantial amounts of resources such as glucose and glutamine and excrete the waste products lactate and ammonia. These alterations apply selective forces that contribute to shaping tumor evolution and the function of stromal cells. A major type of metabolic cooperation in the TME is that of nutrient competition [72]. Tumor-infiltrating lymphocytes are highly glucose-dependent on activation, proliferation and effector function, while on the other hand, they must compete head-to-head with glycolytic cancer cells for this resource [73]. As the availability of glucose decreases, immune cell activity is impaired and therefore promotes immune escape. The same competition also occurs for other metabolites such as glutamine and essential amino acids, necessary for both cancer and stromal cell populations [74]. The result of this competition provides a basis for which cellular subsets are metabolically promoted and functionally repressed in the tumor niche. Besides competition, tumor metabolism is also characterized by metabolic cooperation. Cancer cells may actively reprogram the adjacent stromal cells in order to yield metabolites favorable for tumor support, thus generating a metabolically symbiotic relationship [36]. Well-documented here is the “reverse Warburg effect”, whereby CAFs hijack a glycolytic phenotype and consequently produce lactate, which can then be taken up and oxidized by cancer cells. Cancer cells can also activate autophagy in adjacent stromal cells, thus escalating local amino acid and nutrient availability for the benefit of their own excessive growth [11]. Together, these reciprocal interactions exemplify the bidirectional and flexible metabolic crosstalk that occurs in tumors.

### 5.2. Cancer-Associated Fibroblasts and Metabolic Crosstalk

Cancer-associated fibroblasts (CAFs) are the most abundant stromal cell type in many solid tumors and have diverse roles in promoting tumor progression, including regulating invasive growth, angiogenesis, immune modulation and treatment resistance. Recent evidence has pointed to the metabolic reprogramming of CAFs and their metabolic coupling with cancer cells as major contributors to tumor aggressiveness [64]. Signals provided by tumors, including ROS and transforming growth factor-β (TGF-β), can elicit dramatic metabolic remodeling in fibroblasts characterized by the upregulation of aerobic glycolysis and autophagy to extents that are sometimes even higher than those of cancer cells, remaining committed to oxidative metabolism. The metabolites produced by CAFs are the main nutrients for cancer cells. Lactate produced in fibroblasts via glycolysis can be imported by cancer cells and is utilized in both the TCA cycle for energy (ATP) synthesis and as a biosynthetic intermediate [54]. Autophagy-mediated digestion of cellular substrates in fibroblasts releases amino acids to promote growth and the synthesis of proteins by tumor cells [36]. In addition, in specific conditions, CAFs produce ketone bodies burnt by tumor cells as an alternative energy source. This metabolic division of labor enables spatial and functional segregation within the tumor, which improves global metabolic performance. Aside from nutrient supply, the metabolism of tumors is moderated by CAFs via other pathways. Fibroblasts affect tissue stiffness, nutrient diffusion and cell migration through extracellular matrix deposition. CAFs produce growth factors and cytokines that influence signaling pathways and metabolic programs in cancer and immune cells. Of note, the metabolic phenotype of CAFs is proportional to their ability to promote tumors, as fibroblasts showing high glycolytic activity effectively exert more pro-tumorigenic effects. Indeed, targeting CAF metabolism, or the disturbance of metabolic support led by fibroblasts on tumor cells, has been lately proposed as a shining path to be pursued from a therapeutic standpoint. The major metabolic and immunologic crosstalk in the TME is presented in Table 3.

### 5.3. Metabolism of Immune Cells and Anti-Tumor Immunity

The discovery that immune cell function is closely intertwined with cellular metabolic state has revolutionized the field of anti-tumor immunity and theories behind responses to immunotherapy [85]. Adaptive immune cell populations have unique metabolic programs that are directed towards their function [73]. Upon activation, effector T cells undergo a significant increase in glucose uptake and glycolysis that supports their rapid proliferation and cytokine production, reflecting cancer cell metabolic characteristics. On the other hand, memory T cells preferentially use oxidative phosphorylation along with fatty acid oxidation (a strategy of metabolism associated with durability and long life) [86]. Tregs show another metabolic profile, with increased lipid consumption [87]. Metabolic competition between cancer cells and immune cells has a significant impact on immunity surveillance inside the tumors [72]. Starvation for glucose by avidly glycolytic cancer cells also impairs T cell activation and effector function, contributing to loss of control [73]. Excessive production of lactate also increases immune suppression by acidifying the extracellular environment, leading to downregulation in cytotoxic T cell function, thus promoting the proliferation of immunosuppressive populations, including regulatory T cells and myeloid-derived suppressor cells [54,75]. Sequestration of essential AAs, such as arginine and tryptophan, is another mechanism through which tumors suppress immune responses [78]. Metabolic properties of the TME also affect the efficacy of other immunotherapies, such as immune checkpoint inhibitors [85]. Tumors with high glycolytic rates and severe nutrient deprivation are generally responsive to checkpoint blockade, but the response is often poor, in part because T-cell metabolism and effector function are compromised [88]. On the other hand, interventions aiming to restore the metabolic fitness of immune cells or antagonize immunosuppressive metabolites might improve immunotherapy [74,82]. Accordingly, combination strategies combining immunotherapy with metabolic therapy are actively being pursued [89]. Countering the metabolic limitations enforced by the TME is a key focus and opportunity in contemporary I-O.

### 5.4. Metabolic Signaling and Microenvironment Communication

Metabolites also act as signaling molecules involved in the communication between cells in the tumor microenvironment, not only as driving energy needs and as precursors for biosynthetic purposes. Lactate, traditionally considered a metabolic waste product, acts through the G-protein coupled receptor 81 (GPR81) on various cells, such as cancer, endothelial, and immune cells, to modulate angiogenesis, immunity, and metastasis of cancerous cells [75]. Succinate may act as a signaling molecule via GPR91, thereby influencing immune cell function and angiogenesis. Ketone bodies such as β-hydroxybutyrate also affect histone modifications and gene expression by modulating the activity of histone deacetylases. Metabolites derived from amino acids can also function as signaling molecules. Kynurenine, which is generated during the degradation of tryptophan by indoleamine 2,3-dioxygenase, activates the aryl hydrocarbon receptor and contributes to immunosuppression [78]. The majority of tumors overexpress indoleamine 2,3-dioxygenase, leading to production of the immunosuppressive metabolite kynurenine. Polyamines are synthesized in the context of arginine and ornithine metabolism and modulate several cell processes such as proliferation, differentiation, and immunity. The signaling function of metabolites further adds another level of complexity to tumor microenvironment regulation, forming crosstalk between metabolic and signaling networks that contribute to the modulation of tumor progression and immune responses. Some metabolites are not just substrates or products of metabolism but also extracellular messengers that drive cell behavior in several stromal compartments. One such prominent example is the extracellular adenosine, which accumulates in tumor sites due to ATP release from cells under stress, damaged or dying. Extracellular ATP is converted by the ecto-enzymes CD39 and CD73 to adenosine, which acts on adenosine receptors of both immune and tumor cells. In general, adenosine signaling tends to antagonize anti-tumor immunity by impairing effector T cells but not the tumor survival, eventually promoting neovascularization in addition to immune escape. In line with these findings, CD39 and CD73 are often upregulated in tumor tissues and have emerged as promising therapeutic targets [79]. Pharmacological inhibition of CD73 or adenosine receptor signaling has shown promising anti-tumor effects in preclinical models and is currently being tested in clinical trials, frequently along with antitumoral checkpoint blockers. Collectively, these results demonstrate the intersection of metabolite-mediated signaling with canonical signaling cascades to influence tumor growth and immune suppression.

### 5.5. Acidosis and pH Regulation in Tumors

Many tumors are dependent on accelerated glycolytic activity, which causes overproduction of lactate and protons that can acidify the TME. Thus, the extracellular pH in tumors often drops in the range of 6.5 to 6.9, which is significantly lower than the normal physiological pH of ~7.4. To endure under these conditions, cancer cells activate adaptive mechanisms to maintain intracellular pH homeostasis, such as the upregulation of proton pumps, ion exchangers, and monocarboxylate transporters that remove protons and lactate out of the cell [90]. This apparent counterintuitive pH gradient, of a somewhat alkaline intracellular and an acidic extracellular environment, has diverse biological impacts on tumors. Tumor acidification increases EMT and metastatic potential through extracellular matrix remodeling and protease activation. Acidosis itself changes the distribution and effectiveness of drugs due to pH gradients that drive cell uptake of many cytotoxic agents, especially weak acids or bases. In addition to cancer cells, extracellular acidosis is known to exert an impressive influence on the behavior of both stromal and immune cells. Acidosis inhibits the activation and cytotoxic function of T cells and promotes accumulation and differentiation of immunosuppressive myeloid cell populations [54]. Additionally, acidosis regulates the phenotype of CAFs and activates angiogenic signaling pathways. Moreover, extracellular matrix stiffness and composition are pH-dependent, leading to sub-ideal matrix properties that also impact cell migration and tissue material organization. Together, these effects correlate with tumor acidosis as a master modulator of metabolic, immune and therapeutic responses in the TME. The pH-regulating machinery in cancerous cells can be used as a tool for pharmacological intervention. Carbonic anhydrase IX is overexpressed in various tumors, especially under hypoxia, where it catalyzes the hydration of carbon dioxide into bicarbonate and protons to maintain pH homeostasis. Preclinical models have demonstrated antitumor activity of inhibitors to carbonic anhydrase IX. Monocarboxylate transporters that export lactate/protons are also potential therapeutic targets. Sodium-hydrogen exchangers and vacuolar ATPases that export protons have been studied as potential targets [76,77]. A discordant pH balance could have direct negative effects on cancer cell survival and activate antitumor immunity by alleviating acidosis-related immunosuppression.

## 6. Therapeutic Strategies Targeting Metabolism

### 6.1. Glycolysis Inhibitors

The understanding that numerous cancers operate with increased glycolysis has stimulated extensive efforts to pursue glycolytic inhibitors for cancer treatment [43]. Various enzymes of the glycolytic pathway have been focused on, though with different levels of conviction. 2-Deoxyglucose, a glucose analogue that inactivates both hexokinase and phosphoglucose isomerase, has shown early promise but has been limited by modest single-agent activity and dose-limiting toxicity [91]. Selective inhibitors that focus on individual isoforms of glycolytic enzymes have also been generated in the hope they will be able to produce an improved therapeutic index by leaving normal tissues unexposed. Hexokinase 2 (HK2), a member of the glycolytic pathways highly upregulated in multiple cancer types, has been increasingly demonstrated to be an attractive target. Unlike the other HK isozymes, HK2 is often anchored to the mitochondrial outer membrane and coordinates glucose phosphorylation with ATP generation in the mitochondria while dampening apoptotic signaling. This localization is metabolically energy-efficient and protective against cell death. Experimental agents that inhibit HK2 enzyme activity or prevent its binding to mitochondria have shown substantial anti-tumor effects in preclinical models. However, HK2 is not strictly tumor-specific and is also present in a variety of normal proliferative tissues, which gives rise to valid concerns with off-target toxicity. Therefore, the development of glycolytic inhibitors, which possess even higher tumor selectivity, is still the most significant field for future research [92]. Pyruvate kinase M2 (PKM2) is another glycolytic enzyme that has been broadly investigated and is the isoform cancerous cells preferentially express. PKM2 exhibits distinctive regulation with reversible alternation in the highly active tetrameric form and the low-activity dimeric form. The dimer slows the last step of glycolysis so that the earlier glycolytic intermediates build up and are shunted into pathways that favor macromolecular synthesis and cell growth. Small molecules that bind and stabilize PKM2 in the tetrameric state shift metabolism from biosynthesis toward energy production and suppress tumor growth in vivo. In addition, LDHA therefore not only can convert pyruvate to lactate, but it can regenerate NAD^+^, required for maintaining glycolytic flux as well [91]. While there have been many promising preclinical advances, no compounds that inhibit glycolysis have received regulatory approval for cancer treatment as of yet, highlighting the challenge in specifically targeting metabolic pathways that are also required in normal dividing cells.

### 6.2. Glutaminase and Glutamine Metabolism Targeting

The requirement for glutamine as a carbon and nitrogen donor for the growth of many tumors has driven the development of approaches to target glutamine utilization. Major attention has been paid to glutaminase, an enzyme that initiates the catabolism of glutamine to produce glutamate. CB-839 (telaglenastat), which inhibits the kidney-type isoform GLS1 of glutaminase, has shown potent anti-tumor activity in several preclinical models and is under investigation in various clinical trials [93,94]. Clinical trials have shown CB-839 is generally well tolerated but displays minimal single-agent activity [95]. These findings have raised interest in the use of combinations, driven by understanding of metabolic plasticity. Glutaminase inhibition frequently results in metabolic compensation such as upregulation of glycolysis, autophagy or alternate amino acid uptake by cancer cells [8]. Therapeutic strategies that target shutting down these adaptive pathways result in better anti-cancer responses. For example, the combination of glutaminase inhibitors with autophagy inhibitors or glucose metabolism blockade has demonstrated synergistic activity in preclinical models [11,96]. The increasing use of targeted therapies and immune-based treatments in oncology has recently opened exciting possibilities for the combination with glutaminase blocking agents (Figure 2), which, being a part of multi-modal treatment strategies, adds even more versatility to their initial value as single-agent therapy [74,82]. Outside of glutaminase itself, multiple other nodes of glutamine metabolism offer potential therapeutic inroads. Transporters that mediate glutamine uptake, including ASCT2 and the heterodimer LAT1, have also been exploited using small molecule inhibitors and antibody-based strategies [14]. Additional enzymes that convert glutamate to intermediary TCA cycle products, such as the oxidative deamination of glutamate-by-glutamate dehydrogenase and other aminotransferases, are also candidates. In certain tumor settings, blockade of glutamine synthetase to reduce local glutamine availability might even have an additional detrimental effect on tumor growth. The diversity of targetable nodes in glutamine metabolism provides numerous opportunities for therapeutic intervention, and the optimal target is likely to depend on tumor lineage, metabolic state, and specific genetic lesions [15].

### 6.3. Targeting Amino Acid Dependencies

**Figure 3:**
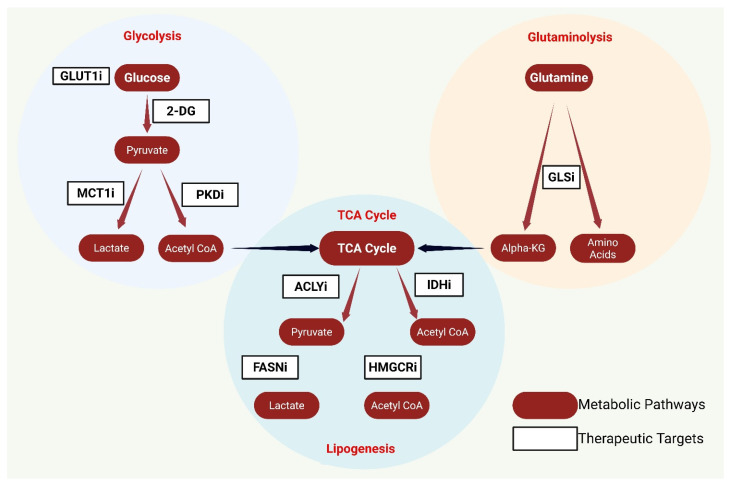
Comprehensive therapeutic targeting maps of tumoral metabolism. The diagram illustrates druggable nodes within the interconnected metabolic network of cancer, encompassing glycolytic enzymes (GLUT1, HK2, PKM2, LDHA, MCT1/4), TCA cycle components (IDH1/2, ACLY), lipogenesis enzymes (FASN, ACC), and glutaminolysis (GLS). Current clinical-stage inhibitors are mapped to their respective targets, illustrating combinatorial therapeutic opportunities. *Created in BioRender. Wali, A. F. (2026). BioRender.com/o4kmmuy.*

As discussed in more detail below, a body of work has suggested that tumors are selectively dependent on some amino acids, leading to metabolic vulnerabilities that may be targeted therapeutically. L-asparaginase that interferes with the extracellular pool of asparagine has been routinely employed for acute lymphoblastic leukemia (ALL) therapy for decades [20]. Asparaginase is effective because most cells express relatively low levels of asparagine synthetase and are dependent on extracellular asparagine. Recent studies have revealed asparagine availability as a factor in the metastatic potential of certain solid tumors, raising the possibility for wider use of asparagine depletion [20,97]. An alternative amino acid-targeted approach is represented by arginine deprivation, which has also been demonstrated to be clinically effective [21]. The expression of argininosuccinate synthetase, the rate-limiting enzyme for synthesizing arginine, has been lost in some cancers such as selected sarcomas, hepatocellular carcinomas and mesotheliomas. Arginine auxotrophic tumors rely on extracellular arginine and are sensitive to the arginine-depleting enzymes pegylated arginine deiminase and arginase [80]. Clinical trials of arginine depletion have demonstrated encouraging evidence of activity against arginine auxotrophic tumors, especially in biomarker-selected strategies [81]. Serine and glycine metabolism represents additional targets of therapy [18]. Phosphoglycerate dehydrogenase inhibitors, the first enzyme in the serine synthesis pathway, are active against cancers where expression of the serine synthesis pathway is increased [30]. Dietary restriction of serine and glycine sensitizes some chemotherapeutics in preclinical models [19]. Methionine restriction (MR), which can be attained through nutritional means or by enzyme depletion, is found to exhibit anti-cancer effects in certain tumor models [22]. These approaches demonstrate how an understanding of tumor-specific amino acid dependencies can be exploited to guide the design of novel therapies and patient selection. The therapeutic targeting landscape of tumor metabolism, which reveals the druggable nodes in glucose, glutamine, amino acid and lipid pathways, is described in Figure 3.

### 6.4. Lipid Metabolism Targeting

The increased lipid biosynthesis found in a variety of cancers, as well as the reliance of certain tumors on fatty acid oxidation, has prompted interest in the targeting of lipids for therapeutic intervention [9]. Inhibitors of fatty acid synthase have been developed and tested in the clinic [28]. TVB-2640, an inhibitor of fatty acid synthase with acceptable tolerability and preliminary signs of activity, particularly in combination with taxanes, has been described [24]. The combination concept is based on the discovery that FAS inhibition impairs microtubule function by affecting tubulin palmitoylation, which may form synergies with taxanes due to their common target (microtubules) [98]. Acetyl-CoA carboxylase, which mediates the committed step in fatty acid synthesis, is another candidate [24]. Acetyl-CoA carboxylase inhibitors, originally developed for metabolic disorders, have anti-cancer effects in preclinical models [24,99]. The two isotypes of acetyl-CoA carboxylase, ACC1 in the cytoplasm for fatty acid synthesis and ACC2 on the mitochondria modulating fatty acid oxidation, represent potential targets for isoform-selective inhibition. Inhibiting ACC2 activates fatty acid oxidation, which may be either a good thing in the case of tumors addicted to it or a bad thing in the case of tumors relying on fatty acid oxidation as an escape route. Stearoyl-CoA desaturase (SCD), which initiates unsaturation in saturated fatty acids, has recently emerged as an attractive metabolic liability in the context of cancer because its suppression induces ferroptosis, an iron-facilitated non-apoptotic cell death associated with lipid peroxidation [25,100]. Inhibition of SCD impacts the organization of membrane lipids and redox homeostasis, leading to oxidative bursts in tumor cells. Cancer cells with certain genetic defects, such as mutations in p53, exhibit particular hypersensitivity to ferroptosis induction, indicative of a genotype-selective therapeutic window [100]. Carnitine palmitoyltransferase 1 (CPT1), the rate-limiting step in mitochondrial fatty acid β-oxidation, is an additional candidate target, especially in cancers with high dependence on lipid oxidation for energy and survival. Such dependence has been observed in subsets of leukemias and in the prostate, where blockade of CPT1 disrupts metabolic homeostasis and cancer growth [27]. The substantial diversity in the lipid metabolic dependencies of cancer subtypes further supports biomarker-based patient stratification to facilitate effective clinical application of lipid metabolism-targeting treatment strategies. Recently, ATP citrate lyase (ACL), which generates cytoplasmic acetyl-CoA from citrate and is a major node that connects glucose metabolism to lipid synthesis and histone acetylation architecture, has been identified as an interesting potential target [49,99]. The compound inhibits ATP citrate lyase, which serves as a rate-limiting enzyme in the lipogenesis pathway, and also reduces histone acetylation, which could provide double-action anti-cancer protection. The clinical development of ATP citrate lyase inhibitors was first performed in the cardiovascular field, but these drugs are now tested for cancer treatment. Other routes of acetyl-CoA production, such as through the use of acetate by acetyl-CoA synthetases, may also confer resistance to ATP citrate lyase inhibition and could serve as additional therapeutic targets [101].

### 6.5. Mitochondrial Targeting Strategies

Notwithstanding the earlier focus on glycolysis as a source of energy or cellular building blocks in cancer, retention of functional mitochondria by most cancer cells and dependency by some cancer cells on glucose oxidation have focused effort on developing mitochondrially targeted anticancer therapeutics [13] (Table 1). Metformin, an oral antihyperglycemic agent for type 2 diabetes that inhibits complex I in the mitochondrial electron transport chain (ETC), exhibits anticancer effects in preclinical models [75,78,85,86,87], and epidemiological studies indicate that use of metformin is associated with decreased incidence of specific cancers in diabetic patients treated with metformin compared to control diabetic patients not receiving such treatment [89,102]. Yet, metformin trials in cancer have given inconsistent findings, which might be due to the relatively moderate complex I inhibition that is attained at tolerable clinical doses. Other, more potent complex I inhibitors, such as IACS-010759, have been developed [13]. IACS-010759 has demonstrated exciting preclinical activity specifically in oxidative phosphorylation-dependent tumors, and it has entered clinical trials [103]. Clinical data to date show acceptable tolerability and early signs of activity in some tumors, such as acute myeloid leukemia. The difficulty with complex I inhibition, just like all other mitochondrial inhibitors, is having enough selectivity for the cancer cells relative to normal cells, which also require functional mitochondria. Complex III, complex V (ATP synthase), and mitochondrial chaperones are also potential MTSs that are currently under study [35,104]. Another possibility is to challenge mitochondrial translation by inhibition of the mitochondrial ribosomes. The mitochondrial protease ClpP is another attractive candidate, as its activation results in selective impairment of mitochondria only in cancer cells. Disparate effects toward cancer cells are evidenced after targeting the mitochondrial dynamics by inhibition of DRP1 or other fission and fusion regulators in preclinical models [59]. The variability in possible mitochondrial targets and the conditional dependence of different cancers on mitochondrial activity point to the necessity for patient stratification using biomarkers in the development of therapies directed against mitochondria.

### 6.6. Combinations and Synthetic Lethality

**Figure 4:**
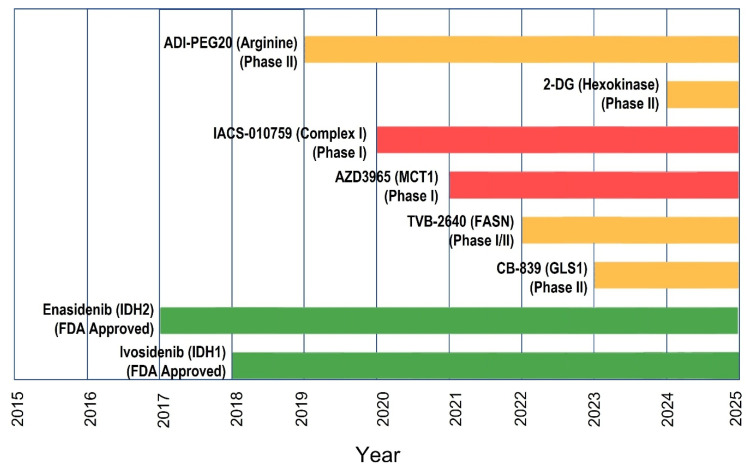
Clinical development timeline of principal metabolic therapeutics (2015–2025). The timeline illustrates the progression of metabolism-directed agents from preclinical validation through phase I–III clinical development, including FDA-approved IDH inhibitors (ivosidenib and enasidenib) and investigational agents targeting glycolysis (2-DG), fatty acid synthesis (TVB-2640), glutaminase (CB-839/telaglenastat), mitochondrial complex I (IACS-010759), MCT1 (AZD3965), and arginine metabolism (ADI-PEG20).

Combination therapy with an HDAC inhibitor and radiotherapy could be superior to either therapy alone [45]. The metabolic flexibility of cancer cells and their ability to induce compensatory pathways in response to single-agent metabolic inhibitors have required the development of rational combination strategies [8]. Combinations might be directed at the same time against two independent metabolic pathways or a metabolic pathway and compensatory mechanisms, or combine metabolism-targeted therapy with other therapeutic strategies. In other words, we want to generate synergistic behavior such that the combination is orders of magnitude more effective than either therapy alone. Some combinatorial approaches are especially promising. In addition, when the inhibition of glycolysis is combined with glutaminase inhibition, cancer cells are obstructed from compensating for their decreased glucose metabolism with elevated rates of glutamine consumption [96]. The combination of metabolic inhibitors and autophagy inhibitors abrogates a significant adaptive response to metabolic stress [11]. The combination of metabolic inhibitors with targeted therapies takes advantage of the heightened metabolic stress by oncogenic pathway inhibition [105]. One example is the combination of inhibitors such as PI3K or mTOR with metabolic blockers, which offers improved therapeutic efficiency relative to the use of each approach on its own [43]. Recently, the concept of collateral sensitivity (i.e., resistance to one agent conferring heightened susceptibility/responsiveness to a second drug) has been extended to metabolic interventions. Cancers that develop resistance to therapy via metabolic reprogramming may be further addicted to their new metabolic situation, for which metabolic inhibitors could exploit their dependency on this altered metabolism [42]. Delineating the metabolic reprogramming that is associated with resistance could inform rational therapeutic sequencing. The identification of prognostic biomarkers capable of predicting the response of patients to metabolic interventions still represents a current challenge for their clinical translation [10]. Figure 4 illustrates the stages in the development of metabolism-based therapeutics from preclinical to clinical studies.

### 6.7. Lessons from the Clinic: Success, Failures and Stratification

The clinical record of metabolism-directed therapies is one of contrast, as it describes both notable successes and several instructive failures (summarized in Table 3 and Figure 4). The primary success is the targeting of mutant isocitrate dehydrogenase, where ivosidenib (IDH1) and enasidenib (IDH2) achieved regulatory approval in IDH-mutant acute myeloid leukemia, and ivosidenib subsequently improved progression-free survival in IDH1-mutant cholangiocarcinoma. These successes share a common trait in which efficacy relies on a specific, testable genetic mutation that generates the actionable oncometabolite driving tumor vulnerability, therefore allowing researchers to treat a genuinely dependent patient population. Conversely, treatments without clear biomarkers have failed. For instance, glutaminase inhibition with telaglenastat (CB-839) was well tolerated by the patients but did not improve progression-free survival in combination regimens in metastatic renal cell carcinoma, likely due to the above-discussed metabolic compensation and the lack of a validated biomarker for glutamine dependence. Similarly, the immunometabolic IDO1 inhibitor epacadostat failed to improve outcomes in advanced melanoma when combined with pembrolizumab, despite a strong mechanistic rationale, demonstrating that preclinical models still poorly predict clinical results. Ultimately, metabolism targets are not inherently flawed, but benefit has been realized only where a predictive biomarker permitted selection of dependent tumors. Without patient selection, tumor heterogeneity and metabolic compensation eroded efficacy. Therefore, future clinical trials must prioritize validated companion biomarkers, rational combination therapies designed to block alternative survival pathways, and resistance-informed sequencing rather than depending on single-agent enzyme inhibitors.

## 7. Critical Insights and Future Directions

Despite a strong mechanistic rationale and consistent preclinical activity, metabolism-targeted agents have, with few exceptions, produced disappointing results in clinical trials, and the recurrent reasons for this gap are largely mechanistic rather than operational. First, the metabolic plasticity that defines cancer also enables rapid compensation, so that inhibition of one pathway is buffered by flux redistribution, as seen when glutaminase inhibition triggers compensatory glycolysis, autophagy, and alternative amino-acid uptake [8,96]. Second, the therapeutic window is intrinsically narrow because proliferating normal tissues share many of the same metabolic dependencies as tumors, limiting tolerable target engagement, a constraint exemplified by complex I inhibition, where dose-limiting toxicity restricts the achievable degree of pathway suppression [103]. Third, intratumoral metabolic heterogeneity means that single-pathway inhibition may clear only a metabolically defined subpopulation while sparing others, permitting outgrowth [10]. Fourth, the field still largely lacks validated predictive biomarkers and pharmacodynamic readouts of target engagement, so trials have frequently enrolled metabolically unselected populations [9,10]. Recognizing these causes reframes the principal knowledge gaps: how to predict the compensatory route a given tumor will take, how to identify the patients whose tumors are genuinely dependent on a targeted node, and how to widen the therapeutic window through tumor-selective delivery or synthetic-lethal combinations.

### 7.1. Technologies Reshaping the Field

Cancer metabolism has been catalyzed by revolutionary technologies. Stable isotope tracing with ^13^C labeled nutrient supply and mass spectrometry-based metabolomics permits direct quantitative determinations of metabolic fluxes such that unanticipated requirements and pathway utilizations are accessible when steady-state analyses do not have sufficient resolution [3]. In vivo application of these methodologies, in both patient-derived tumors and normal tissue, reveals new insights into cancer metabolism within a more physiologically relevant environment. Spatial metabolomics methodologies can image metabolites in intact tissues, revealing dramatic intra-tumoral metabolic heterogeneity [10]. Imaging techniques based on mass spectrometry, including MALDI and DESI, allow several hundred metabolites to be observed at the subcellular level. Complementarily to spatial transcriptomics and proteomics, OmniFISH gives a more all-round view of the interplay between gene expression, protein concentration and metabolic state [71]. Single-cell methodologies are technically demanding but provide key insights into metabolic diversity. Methods such as mass cytometry, Raman spectroscopy and fluorescent biosensors can assess the metabolic states of individual cells, and sequencing techniques—from RNA (single-cell RNA seq) to protein (cytometry by time of flight; CIBER-seq3)—provide more indirect information via RNA expression of metabolically relevant enzymes or transport proteins. When combined with single-cell data, this approach has the potential to uncover how genetic heterogeneity shapes metabolic diversity and cell fates.

The complexity of metabolic networks has led to the development of computational approaches aimed at modeling and predicting metabolic dynamics [106]. Constraint-based modeling approaches, such as flux balance analysis, determine metabolic fluxes on the basis of stoichiometric and optimization principles. GSMMs offer an opportunity for systematic discovery of vulnerabilities and can be tailored with tumor-specific omics data. Kinetic models include regulation and kinetics of enzymes to facilitate an even more accurate prediction of perturbation effects. Metabolomics, leveraging machine learning methods, allows identification of patterns that predict drug response and patient outcome. Computational analysis of metabolic imaging and stable isotope tracing data helps to extract relevant biological information from spatial as well as flux data. With the growth of metabolic datasets, computational integration is required for target prioritization and hypothesis formulation. However, despite the preclinical excitement, metabolic therapies have encountered translational hurdles [9]. Metabolic plasticity of cancer allows activation of compensatory pathways due to similarities between cancer and normal proliferating cells, which is a cause for concerns about toxicity [8]. Tumor metabolic heterogeneity could facilitate the escape of resistant subpopulations from single-agent inhibitors [10]. The absence of predictive biomarkers adds to the complexity of their clinical translation. Approaches that have been proposed to address these challenges include the design of rational combinations to curb adaptive responses [11] and biomarker development for metabolic dependency or target engagement [10]. Incorporation of metabolic imaging with PET and MR spectroscopy in trials may also allow a non-invasive, dynamic assessment of response to therapies. Drug repurposing with agents such as metformin, statins and FAS inhibitors provides an opportunity to fast-track clinical translation; however, oncology-specific dosing may contrast with indications for these agents on metabolic indication [89,102,107].

### 7.2. The Personalized Metabolic Medicine Imperative

In view of the inter- and intratumoral metabolic heterogeneity, individualized modalities are necessary [10]. Metabolomics of tissue or liquid biopsies, functional ex vivo assays and metabolic imaging can be used to identify patient-specific vulnerabilities. Integrating with genomic and transcriptomic data via multi-omics approaches, metabolic therapies can be matched to tumor-specific dependencies [106]. Some genetic alterations, IDH mutations or MTAP deletion, are predictive for metabolic sensitivities [108]. Guidelines for personalized metabolic therapy: Patients should be treated according to individual biochemical phenotype, which can be used to establish personalized patient metabolic profiles allowing selection of appropriate paths of OMT for therapy. Hopefully, future clinical decision support tools will help in the integration of multi-omics data.

### 7.3. Unresolved Controversies and Priority Research Questions

Crucial questions that loom involve the underlying causes of glycolytic versus oxidative phenotypes among tumors [5,13], to what extent these reflect genetics versus microenvironment [33], and what functional implications metabolic heterogeneity has for therapeutic resistance [10]. It remains important to understand metabolic dependencies throughout the spectrum of tumor cells from initiation to metastasis as well as in resistant populations, including CSCs and dormant cells [20,57]. In addition, the association between metabolism and other cancer hallmarks—genome instability, immune evasion or therapy resistance—across these two temporal and molecular scales requires further investigation [7,72,85]. Elucidation of the mechanisms by which metabolic adaptations impact mutation rates, DNA repair, immune responses and therapy resistance is necessary for successful translation.

## 8. Conclusions

In the past decade, tumor metabolism has shifted from backwater to the central dogma of cancer biology. Metabolic reprogramming is a major hallmark of malignancy and impacts tumor proliferation, survival, redox homeostasis and response to therapy. Although establishing the Warburg effect is of historic importance, modern understanding includes glucose, amino acid, lipid and one-carbon metabolism as well as retained and repurposed mitochondrial function and dynamic TME interactions. Mechanistic understanding of how oncogenic drivers, loss of tumor suppressors, hypoxia, and alterations to the epigenetic landscape intersect can be elucidated from these studies to reprogram metabolism. PTM, metabolite signaling and metabolism–epigenetics crosstalk operates at various levels to establish intricate regulatory hubs that underpin fast metabolic adaptation. The tumor microenvironment is key to metabolic behavior, with cancer and stromal cells competing and cooperating to drive progression and treatment response, metabolic checkpoints, immune function and immunotherapy synergy opportunities. Despite difficulties, translation of metabolic findings into rational therapies is possible and currently ongoing. Rational combinations, predictive biomarkers, repurposed metabolic drugs and personalized metabolic profiling are leading to the path of precision metabolic medicine. Integration of multi-omics data with state-of-the-art imaging will continue to optimize patient selection and prioritize therapeutic approaches. Finally, the vulnerabilities created by the metabolic stresses of unrestrained proliferation and the local tumor microenvironment also offer themselves as pickable Achilles’ heels. Complemented by ongoing advances, metabolism-targeted anticancer therapies should emerge as a revolutionary part of precision oncology over the next few decades.

## Figures and Tables

**Table 1:** Predominant metabolic phenotypes and representative therapeutic vulnerabilities across major cancer types.

Cancer Type	Glycolytic Index	OXPHOS Dependence	Metabolic Plasticity	Key Metabolic Features	Primary Therapeutic Vulnerabilities	Refs.
Glioblastoma	High	Low-Moderate	Moderate	Glucose/glutamine co-dependence, IDH mutations in secondary GBM,	IDH inhibitors, mTOR inhibitors, glutaminase inhibition + autophagy blockade	[17,34,35]
Pancreatic adenocarcinoma	High	Low	High	Autophagy, macropinocytosis, amino acid scavenging, KRAS-driven	Autophagy inhibitors, micropinocytosis blockade, stromal targeting, KRAS (G12C/D) inhibitors + metabolic	[8,11,36]
Renal cell carcinoma	Variable	High	High	Lipid accumulation, constitutive HIF activation (VHL loss), glucose/glutamine	CAIX inhibitors, OXPHOS inhibitors, combination metabolic strategies (CB-839 monotherapy failed)	[16,37]
Acute myeloid leukemia	Moderate	High	High	Glutamine/leucine dependence, OXPHOS reliance, IDH mutations, BCL-2 dependance	IDH1/2 inhibitors (ivosidenib/enasidenib), venetoclax + OXPHOS inhibitors, IACS-010759	[13,34,38,39]
Breast cancer (ER+)	Moderate	Moderate-High	High	Fatty acid oxidation, estrogen-regulated metabolism	ACC inhibitors, FASN inhibitors, PI3K/mTOR + metabolic combination	[26,40,41]
Lung adenocarcinoma	High	Low-Moderate	Moderate	KRAS-driven glutaminolysis, glucose addiction	KRAS (G12C) inhibitors (sotorasib/adagrasib) + metabolic; NRF2 pathway inhibitors	[10,33,42]
Melanoma	Moderate-High	Moderate	High	BRAF-regulated metabolism, metabolic flexibility	BRAF/MEK + OXPHOS inhibitors, ferroptosis inducers (GPX4/SCD inhibitors) in selected patients	[4,7]
Colorectal cancer	High	Moderate	Moderate	WNT-driven glycolysis, glutamine metabolism	Glycolysis inhibition (LDHA, HK2), glutaminase inhibitors (CB-839), WNT/β-catenin pathway targeting, anti-angiogenic + metabolic therapy (bevacizumab combinations), OXPHOS inhibitors	[2,4]

**Table 2:** Metabolic–epigenetic interactions in cancer: mechanisms and therapeutic implications.

Metabolite	Source Pathway	Epigenetic Target	Function	Therapeutic Relevance	Refs.
α-Ketoglutarate (α-KG)	TCA cycle	TET, JmjC demethylase	Cofactor	IDH inhibitors restore levels	[17,34]
2-Hydroxyglutarate (2-HG)	Mutant IDH	TET, JmjC demethylase	Inhibitor	Oncometabolite direct target	[17,34,52]
Acetyl-CoA	Multiple	HATs	Cofactor	Links metabolism to transcription	[49,50,53]
S-Adenosylmethionine (SAM)	One-carbon	DNMTs, HMTs	Cofactor	Methionine cycle targeting	[22,29]
Succinate	TCA cycle	TET, JmjC demethylase	Inhibitor	Accumulation in SDH mutants	[48]
Fumarate	TCA cycle	TET, JmjC demethylase	Inhibitor	Accumulation in FH mutants	[48]
Lactate	Glycolysis (LDHA)	Histone lactylation (H3K18la)	Epigenetic modifier	Metabolic–epigenetic crosstalk, novel target for gene expression regulation	[51,54]

**Table 3:** Metabolic immune interactions in tumor microenvironment: mechanisms and clinical experience.

Metabolic Factor	Source	Mechanism	Immune Impact	Therapeutic Target	Clinical Status	Refs.
Lactate (10–30 mM)	Tumor glycolysis	Acidification, MCT1 competition, signaling	T cell inhibition, Treg promotion, M2 polarization	MCT1/4 inhibitors (AZD3965)	Phase I/II	[54,75,76,77]
Kynurenine	IDO1 activity	Trp depletion (GCN2 activation), AhR signaling	T cell suppression, Treg activation	IDO1 inhibitors (epacadostat)	Failed Phase III (ECHO-301)	[78]
Adenosine	CD39/CD73 pathway	A2A receptor signaling (↑cAMP)	Broad immunosuppression	CD73 inhibitors (oleclumab)	Phase I/II (ongoing)	[79]
Arginine depletion	Arginase (MDSCs)	Protein synthesis block (T cells lack ASS1)	T cell dysfunction	Arginase inhibitors (CB-1158)	Phase I/II	[80,81]
ROS	MDSCs, TAMs	Oxidative damage to TCR, signaling disruption	T cell dysfunction	Antioxidants, MDSC depletion	Preclinical	[7]
Glutamine depletion	Tumor consumption	Metabolic stress	Context-dependent (may impair T cells or tumor)	Glutaminase inhibitors + immune	Failed Phase III (CANTATA)	[74,82]
PGE2	COX-2/mPGES-1	EP2/EP4 receptor signaling	Immunosuppression, Treg promotion	COX-2 inhibitors, EP4 antagonists	Preclinical/ Phase I/II	[83,84]

## Data Availability

No new data were created or analyzed in this study. Data sharing is not applicable to this article.

## Abbreviations

The following abbreviations are used in this manuscript:

2-DG	2-Deoxyglucose
2-HG	2-Hydroxyglutarate
ACC	Acetyl-CoA carboxylase
ACLY	ATP-citrate lyase
AhR	Aryl hydrocarbon receptor
AMPK	AMP-activated protein kinase
ASS1	Argininosuccinate synthase 1
ATP	Adenosine triphosphate
CAF	Cancer-associated fibroblast
COX-2	Cyclooxygenase-2
CSC	Cancer stem cell
DC	Dendritic cell
DESI	Desorption electrospray ionization
DNMT	DNA methyltransferase
DRP1	Dynamin-related protein 1
EMT	Epithelial–mesenchymal transition
ETC	Electron transport chain
EV	Extracellular vesicle
FAO	Fatty acid oxidation
FASN	Fatty acid synthase
FH	Fumarate hydratase
GLS1	Glutaminase 1 (kidney-type)
GPR81	G-protein coupled receptor 81
GPX4	Glutathione peroxidase 4
GSMM	Genome-scale metabolic model
HAT	Histone acetyltransferase
HDAC	Histone deacetylase
HIF	Hypoxia-inducible factor
HK2	Hexokinase 2
HMT	Histone methyltransferase
IDH	Isocitrate dehydrogenase
IDO1	Indoleamine 2,3-dioxygenase 1
JmjC	Jumonji C domain-containing demethylase
LDHA	Lactate dehydrogenase A
LKB1	Liver kinase B1
MCT	Monocarboxylate transporter
MDSC	Myeloid-derived suppressor cell
mTOR	Mechanistic target of rapamycin
MFN	Mitofusin
mtDNA	Mitochondrial DNA
NADPH	Nicotinamide adenine dinucleotide phosphate
NRF2	Nuclear factor erythroid 2-related factor 2
OPA1	Optic atrophy protein 1
OXPHOS	Oxidative phosphorylation
PGC-1α	PPAR gamma coactivator 1-alpha
PGE2	Prostaglandin E2
PHGDH	Phosphoglycerate dehydrogenase
PI3K	Phosphoinositide 3-kinase
PKM2	Pyruvate kinase M2
PTEN	Phosphatase and tensin homolog
PTM	Post-translational modification
RAS	Rat sarcoma protein
RB	Retinoblastoma protein
ROS	Reactive oxygen species
SAM	S-adenosylmethionine
SCD	Stearoyl-CoA desaturase
SDH	Succinate dehydrogenase
TAM	Tumor-associated macrophage
TCA	Tricarboxylic acid cycle
TET	Ten-eleven translocation
TIGAR	TP53-induced glycolysis & apoptosis regulator
TME	Tumor microenvironment
TnT	Tunneling nanotube
Treg	Regulatory T cell
UPR	Unfolded protein response
VHL	Von Hippel-Lindau tumor suppressor
